# Difficult Airway Management in the Intensive Care Unit: A Narrative Review of Algorithms and Strategies

**DOI:** 10.3390/jcm14144930

**Published:** 2025-07-11

**Authors:** Talha Liaqat, Mohammad Asim Amjad, Sujith V. Cherian

**Affiliations:** 1 Internal Medicine, Wright Center for Graduate Medical Education, Scranton, PA 18510, USA; liaqatt@thewrightcenter.org; 2 Division of Critical Care, Pulmonary and Sleep Medicine, University of Texas Health Science Center, Houston, TX 77030, USA; sujith.v.cherian@uth.tmc.edu

**Keywords:** difficult airway, intubation, airway, bougie, laryngoscopy, cricothyrotomy

## Abstract

**Background:** The management of difficult airways is one of the most critical and challenging aspects of emergency and ICU care. Despite technological advances, unanticipated airway difficulty can result in serious complications, including hypoxia, brain injury, and death. This comprehensive narrative review aims to consolidate current algorithms and evidence-based strategies to guide clinicians in the assessment and management of difficult airways. **Methods:** A comprehensive literature review was conducted using PubMed, Embase, and Google Scholar to identify relevant studies, clinical guidelines, and expert consensus documents related to difficult airway management. The focus was placed on both pre-intubation assessment tools and intervention strategies used in various clinical contexts. **Results:** Airway difficulty is best anticipated through a combination of history, physical examination, and validated tools such as the Mallampati score. Several algorithms, including those from the American Society of Anesthesiologists (ASA) and the Difficult Airway Society (DAS), provide structured approaches that emphasize preoxygenation, preparedness for failed intubation, and the use of adjuncts such as video laryngoscopy, supraglottic airway devices, and awake intubation techniques. Crisis algorithms such as the Vortex approach help simplify decision-making during emergencies. It is important to have adjuncts available in cases of anticipated difficult airways, such as fiberoptic intubation, while surgical airway access is an important component of a stepwise airway management algorithm when critical scenarios are encountered. **Conclusions:** Effective difficult airway management requires anticipation, a structured plan, familiarity with advanced airway tools, and adherence to validated algorithms. Training in crisis resource management and multidisciplinary rehearsal of airway scenarios are essential to improving outcomes.

## 1. Introduction

Managing difficult airways is one of the most important challenges in critical care and emergency medicine. Failure to do so in a timely manner can result in respiratory arrest and consequent hypoxic brain injury within minutes, making airway management a top priority in the intensive care unit (ICU). A “difficult airway” is generally defined as a situation in which a trained clinician encounters difficulty with face mask ventilation, tracheal intubation, or both. According to the 2022 American Society of Anesthesiologists (ASA) guidelines, a difficult airway may involve difficulty in facemask ventilation, supraglottic airway use, intubation, or a complex combination of these factors [1]. ICU patients often present additional challenges—they may be obtunded, agitated, or have physiologic derangements (e.g., severe hypoxemia, acidosis, or RV failure; defined as the inability of the right ventricle to generate adequate forward flow to maintain pulmonary circulation and left ventricular preload) that make standard airway management more hazardous. This concept of the “physiologically difficult airway” has gained attention, emphasizing that even when anatomy is normal, critical illness (e.g., shock or respiratory failure) can create a difficult airway scenario due to rapid desaturation or cardiovascular collapse during intubation [2].

Multiple professional organizations have developed algorithms and guidelines to improve airway management safety. The ASA released updated difficult airway guidelines in 2022, building on prior versions, to incorporate new tools like video laryngoscopy and highlight early identification and planning [1]. The Difficult Airway Society (DAS) in the UK issued its 2015 guidelines focusing on unanticipated difficult intubation in adults, structured around a clear plan from initial intubation attempts through emergency surgical access [3]. Similarly, the Intensive Care Society and DAS collaborated on 2018 guidelines specifically for managing ICU intubations, acknowledging unique ICU challenges and stressing the importance of first-pass success and avoidance of hypoxia [4]. The French Society of Anesthesia and Intensive Care released its own guidelines (2018) reinforcing many of these principles in the French healthcare context [5]. Even critical care and respiratory societies (e.g., CHEST and the American Thoracic Society) have published recommendations relevant to airway management in critical illness [6]. Despite these guidelines, securing an airway in the ICU remains high-risk. The Fourth National Audit Project (NAP4) in the UK highlighted that airway complications in the ICU and emergency departments were far more likely to result in death or brain damage than those in the operating theater. In fact, NAP4 reported that a significant proportion of major airway events in the ICU (approximately 60% of cases) led to patient death or severe neurological injury, compared to much lower rates in elective surgical settings [7]. This stark finding underscored gaps in preparation and training for difficult airways outside the OR environment. More recently, the INTUBE study (2021), a 29-country prospective investigation of ICU intubations, found that 45.2% of these patients experienced at least one major adverse event within 30 min of tracheal intubation [8].

Given the high stakes, a well-structured approach with a backup plan is often warranted when a difficult airway is anticipated. This means mobilizing not just the intensivist or anesthesiologist, but also nursing staff, respiratory therapists, and sometimes surgical specialists (for backup surgical airway) to be immediately available. Many institutions now employ pre-intubation checklists to ensure that all necessary equipment is prepared and that the team is ready for possible difficulty [9].

This narrative review provides a comprehensive overview of difficult airway management in critical care, combining evidence-based algorithms with practical clinical insights for real-world use. We will discuss methods to evaluate and predict a difficult airway, detailed strategies to secure an airway in the ICU (ranging from video laryngoscopy to surgical cricothyrotomy), and an algorithmic approach to decision-making. The objective of this review is to synthesize established guidelines with frontline clinical practice, offering a structured, pragmatic framework to help clinicians anticipate, prepare for, and manage airway challenges. By systematically assessing risk factors and following established algorithms, clinicians can mitigate airway complications and ensure the patient’s oxygenation and ventilation are maintained [2].

## 2. Methods

This manuscript is presented as a narrative review synthesizing both evidence-based recommendations and practical strategies for the management of the difficult airway in critically ill adult patients. We conducted a comprehensive literature review using multiple databases, including PubMed, MEDLINE, and Google Scholar. Search terms included combinations of “difficult airway,” “ICU intubation,” “emergency airway,” “video laryngoscopy,” “fiberoptic intubation,” “rapid sequence induction,” “supraglottic airway,” “bougie,” “cricothyrotomy,” “sedation for intubation,” “airway algorithm,” and “airway complications.” Boolean operators (AND, OR) were used to expand or narrow results based on thematic relevance.

We included clinical practice guidelines (e.g., American Society of Anesthesiologists [ASA] 2022 and Difficult Airway Society [DAS] 2015), randomized controlled trials, observational studies, meta-analyses, and expert consensus statements relevant to adult airway management in critical care. Foundational physiologic principles and key historical trials were also referenced when appropriate, particularly in sections covering pharmacology and airway anatomy. All of the included literature was reviewed for relevance to adult critically ill populations, particularly in the setting of emergency or urgent airway management.

## 3. Airway Assessment and Prediction of Difficulty

A thorough airway assessment is the first step in formulating an airway management plan. The goal is to identify patients at risk for difficult ventilation or intubation before induction of anesthesia or loss of protective reflexes. According to ASA guidelines, a difficult airway can manifest in several ways: difficult face mask ventilation (inability to maintain oxygenation with a mask), difficult supraglottic airway placement or ventilation, difficult laryngoscopy (inability to visualize the vocal cords), difficult intubation (requiring multiple attempts or special techniques), or failed intubation [1]. Each of these potential difficulties should be considered during the pre-intubation assessment.

### 3.1. Predictive Factors

Numerous bedside assessments and predictors have been studied to foresee a challenging airway. No single exam finding perfectly predicts difficulty, but combinations of factors increase suspicion. A 2019 systematic review by Detsky et al. evaluated the accuracy of common bedside screening tests for difficult intubation. They found that while many tests have limited sensitivity individually, some are more useful in ruling out a difficult airway. For example, the Mallampati classification (visualizing oropharyngeal structures with the patient sitting, mouth open, tongue protruded) can suggest difficulty if it is Class III or IV (minimal or no uvula/palatopharyngeal wall visible). Mallampati Class III/IV is associated with a higher incidence of difficult laryngoscopy, though on its own it has a moderate positive predictive value [10].

**Figure 1:**
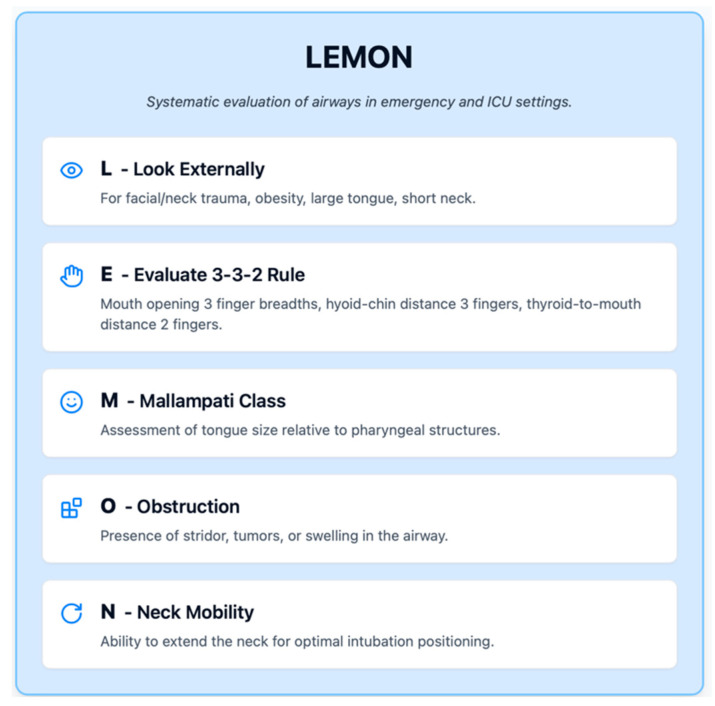
LEMON mnemonic for evaluating and predicting difficult airway.

In emergency and ICU settings, the mnemonic “LEMON” (Figure 1) is often used to systematically evaluate airways: Look externally (for facial/neck trauma, obesity, large tongue, or short neck), Evaluate 3-3-2 rule (mouth opening three finger breadths, hyoid–chin distance three fingers, and thyroid-to-mouth distance two fingers), Mallampati class, Obstruction (stridor, tumors, and swelling), and Neck mobility. While developed for trauma and emergency intubations, LEMON covers key elements relevant in ICU patients as well.

**Figure 2:**
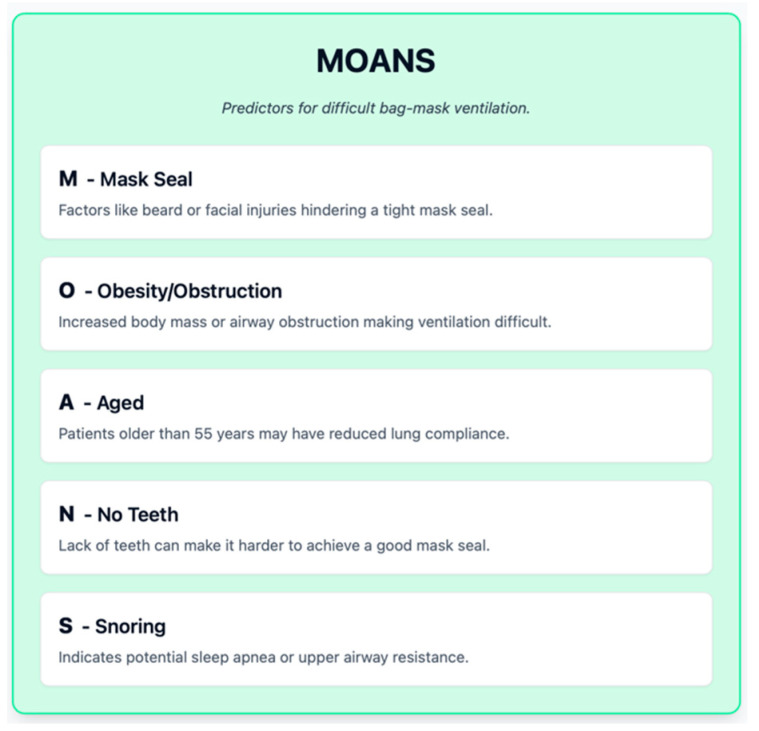
The MOANS mnemonic for predicting difficult bag-mask ventilation.

For difficult bag-mask ventilation, predictors can be remembered by “MOANS” (Figure 2): Mask seal (e.g., beard or facial injuries), Obesity/Obstruction, Aged (older than 55), No teeth, Snoring (or sleep apnea).

**Figure 3:**
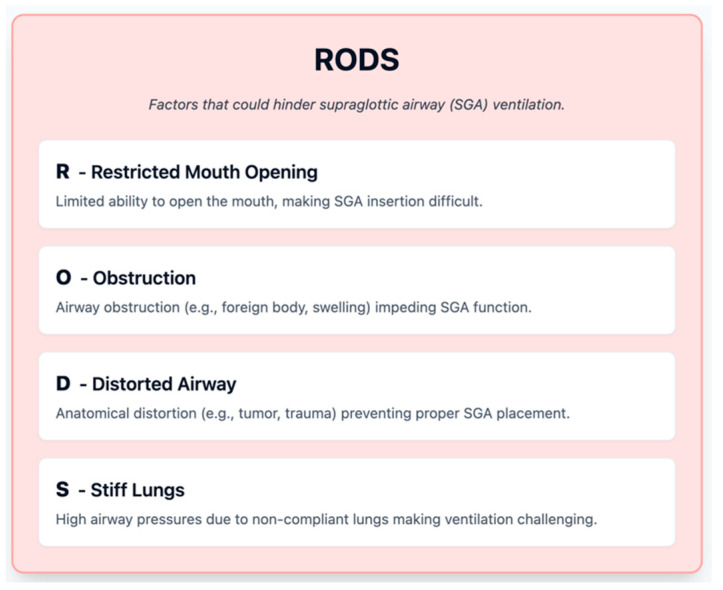
The RODS mnemonic for predicting difficulty with supraglottic airway placement.

For difficult supraglottic airway use, the “RODS” mnemonic (Figure 3) highlights Restricted mouth opening, Obstruction, Distorted airway (e.g., tumor), and Stiff lungs (high airway pressures) as factors that could hinder SGA ventilation. These assessments help anticipate not only intubation difficulty but also whether a backup oxygenation method like a Laryngeal Mask Airway (LMA) might fail.

### 3.2. Scoring Systems

In ICU patients, who often differ from elective surgical populations, specialized predictive scores have been developed. One widely cited tool is the MACOCHA score, created by a French ICU team. A MACOCHA score of 3 or more was found to predict difficult intubation in ICU patients with good sensitivity and specificity in the initial study [11]. For example, a comatose obese patient with OSA who has a Mallampati classification of III and is being intubated by a non-anesthesiologist would score high, alerting the team to a high-risk airway. While MACOCHA is a useful risk stratification tool, clinicians should use it to augment clinical judgment, not replace it. It prompts attention to key risk factors particularly relevant in the ICU context (e.g., the intubator’s experience level and the presence of severe hypoxemia, which can quickly complicate intubation).

### 3.3. Airway Examination

A focused physical exam is critical. Key elements include the following:

- Mouth Opening: Assess the inter-incisor distance (normal >4 cm). Limited mouth opening (trismus, TMJ issues, or edema) may preclude direct laryngoscopy or even video laryngoscope insertion. It also makes placement of a bite block or LMA difficult.
- Tongue and Dentition: A large tongue relative to oral cavity (as in Mallampati III/IV) can obstruct the view. The presence of loose or protruding teeth is noted (to avoid dental trauma and as a clue to potentially narrow oral space). Dentures should typically be removed right before intubation (they improve face-mask seal if left until just prior to laryngoscopy).
- Jaw Protrusion: Ask the patient (if awake) to slide the lower jaw forward or bite the upper lip. If they cannot protrude the lower incisors past the upper incisors, it may indicate reduced subluxation capability and a more difficult laryngoscopic view.
- Neck Range of Motion: Observe flexion and extension of the neck. Conditions like cervical spine immobilization (e.g., trauma with C-collar), severe arthritis (ankylosing spondylitis), or prior cervical fusion can severely limit alignment of the oral–pharyngeal–laryngeal axes, making intubation challenging.
- Thyromental Distance: Measure (with fingers or a ruler) the distance from the chin (mentum) to the top of the thyroid cartilage with the neck extended. Less than about 6 cm (three ordinary finger breadths) is considered a short thyromental distance, indicating a potentially anterior larynx that is hard to visualize.
- Cricothyroid Membrane: Palpate the neck to identify landmarks (hyoid, thyroid notch, and cricoid cartilage). Difficult or impossible palpation of the cricothyroid space (due to obesity or neck mass) can predict difficulty if an emergency cricothyrotomy is needed [12].

### 3.4. When to Consider an Awake Intubation

If the airway assessment reveals multiple red flags suggesting that after induction you might not be able to intubate or ventilate the patient, an awake intubation is usually the safest course. Awake intubation allows the patient to maintain their own oxygenation and airway reflexes during the intubation attempt, theoretically preventing the “cannot intubate, cannot ventilate” crisis. ASA guidelines strongly advocate awake intubation when both intubation and ventilation are anticipated to be difficult [1]. Examples include patients with severe facial or neck abnormalities, high risk of obstruction (like a large lingual thyroid or epiglottic abscess), or extreme obesity with expected rapid desaturation. In practice, awake intubation in ICU can be challenging due to lack of patient cooperation (if delirious or encephalopathic)—so early consideration of this approach and adequate topical anesthesia/sedation is required to make it successful.

In summary, airway assessment in critical care should be systematic and thorough. Using a combination of history, exam, and risk scores like MACOCHA improves identification of a difficult airway [11]. No predictive method is foolproof; studies indicate that unanticipated difficult intubations still occur even when assessments are done. For instance, a meta-analysis by Shiga et al. noted that the absence of any risk factor does not guarantee an easy airway—sensitivity of most tests is in the 20–60% range [13]. Therefore, clinicians must always be prepared with a backup plan even if the airway appears easy. Every intubation in ICU should be approached with a difficult airway mindset.

## 4. Preparation and Optimization for Intubation

Once a potentially difficult airway is recognized, exhaustive preparation is crucial. Preparation can be conceptualized in terms of equipment, patient optimization, team preparation, and backup planning.

### 4.1. Equipment and Environment

A fundamental principle is to have a standard difficult airway cart or kit in the ICU stocked with advanced airway devices: various laryngoscope blades (Macintosh and Miller), at least one video laryngoscope with assorted blade sizes, supraglottic airways of different sizes, a flexible fiberoptic bronchoscope, intubating stylets and bougies, cricothyrotomy kits, and other adjuncts (magill forceps, tape, end-tidal CO_2_ detector, etc.). Before intubation, especially a high-risk one, the intubating clinician should visually confirm that all needed devices are present and functioning. Checking the equipment includes testing the laryngoscope light or video monitor, ensuring the endotracheal tube cuffs hold pressure, and having suction working and immediately at hand. The environment should be optimized: adequate lighting, bed at proper height, and ideally the patient positioned such that the intubator has room to maneuver. If possible, remove part of the ICU bed headboard or any obstacles that could hinder positioning for intubation.

### 4.2. Patient Positioning

Proper positioning can significantly improve airway management success. The classic “sniffing position” (neck flexed and head extended) aligns the oral, pharyngeal, and laryngeal axes for direct laryngoscopy. In practice, this can be achieved by elevating the head about 7–10 cm with blankets or a foam pillow, such that the external auditory meatus is horizontally aligned with the sternal notch (this is often referred to as ear-to-sternal-notch positioning). In obese patients, a ramped position is recommended: multiple blankets or a special ramp device is used to elevate the upper back, neck, and head, forming a slope that aligns the ear and sternal notch and helps prevent the pannus from pressing on the diaphragm. Ramping an obese patient can markedly improve laryngoscopic view and delay desaturation by improving FRC (functional residual capacity) when supine.

For patients in respiratory failure who are on noninvasive ventilation or high-flow nasal cannula prior to intubation, intubating in a semi-upright position can help with preoxygenation and may reduce the risk of aspiration. There is some evidence that intubating in a 25–30-degree head-up position, rather than completely supine, can prolong safe apnea time and improve intubation conditions in obese or severely hypoxemic patients [14]. Thus, unless contraindicated (e.g., unstable spinal injury), elevating the head of the bed during airway preparation and preoxygenation is strongly recommended. Head-of-bed elevation at 20–45° has been shown to improve functional residual capacity, reduce atelectasis, and enhance preoxygenation by delaying desaturation during apnea, especially important in obese or critically ill patients. While the bed can be returned to flat if necessary during laryngoscopy, many experienced practitioners intubate successfully with the bed partially elevated, even during direct or video laryngoscopy [14].

### 4.3. Preoxygenation

Adequate preoxygenation is perhaps the single most important step to prevent desaturation during difficult airway management. Preoxygenation aims to replace the nitrogen in the lungs with oxygen, creating an oxygen reservoir in the functional residual capacity that can extend the time to critical desaturation during apnea. The traditional method is to administer high-concentration oxygen for 3–5 min of normal tidal breathing through a tight-fitting face mask. In ICU patients, many have shunt physiology or diffusion issues that make preoxygenation less effective. Strategies to maximize oxygenation include using noninvasive positive-pressure ventilation (NIV) for preoxygenation, adding positive end-expiratory pressure (PEEP) to the breathing system, and using high-flow nasal oxygen concurrently.

Studies have shown that preoxygenation with noninvasive ventilation (e.g., BiPAP) can significantly improve oxygen saturations in hypoxemic patients compared to a non-rebreather mask. Baillard et al. (2006) demonstrated in a randomized trial that patients with severe hypoxemia (e.g., due to acute lung injury) who received preoxygenation via NIV (pressure support with PEEP) had higher oxygen tensions and were less likely to desaturate during intubation than those breathing spontaneously on a reservoir face mask [15]. The PEEP provided by NIV helps recruit alveoli and counteract atelectasis, thus preventing oxygen desaturation. Based on such evidence, many ICU protocols incorporate 3–5 min of NIV (with an FiO_2_ of 1.0 and 5–10 cm H_2_O of PEEP) for any patient who can tolerate it prior to intubation, especially if their pre-intubation SpO_2_ on oxygen is low. If NIV is not an option (e.g., the patient cannot tolerate the mask or there is no time), a tight seal with a bag-valve-mask and 15 L/min O_2_ (plus possibly an in-line PEEP valve set to 5–10) can be used to preoxygenate.

The high-flow nasal cannula (HFNC) is another powerful tool for preoxygenation. A HFNC can deliver heated humidified oxygen at flow rates up to 60 L/min, with FiO_2_ approaching 100%. It provides a degree of apneic oxygenation due to the continuous flow. Patel and Nouraei (2015) described the THRIVE technique—essentially using high-flow nasal oxygen to prolong safe apnea time—and reported markedly lengthened apnea tolerance in obese patients [16]. In the ICU, a high-flow nasal cannula can be placed on the patient for several minutes before intubation to wash out nitrogen. Importantly, a HFNC can be left in place during laryngoscopy to provide apneic oxygenation throughout the intubation attempt. This continuous oxygen delivery can postpone desaturation even when the patient is not spontaneously breathing. A randomized trial by Frat et al. (2019)—comparing a HFNC vs. noninvasive ventilation for preoxygenating ICU patients—found that both methods were effective, and in certain hypoxemic patients, a HFNC was non-inferior to NIV in preventing desaturation [17].

Another adjunct is apneic oxygenation with nasal cannula—even a standard nasal cannula at 15 L/min can provide apneic oxygenation if left in place during intubation attempts. The FELLOW trial (Semler et al. 2016) tested apneic oxygenation with a 15 L nasal cannula in critically ill adults and found that while it did not significantly increase lowest oxygen saturations compared to no apneic oxygenation, it is a low-risk intervention that many still employ [18]. Essentially, apneic oxygenation is a safety net that may help a little and is unlikely to harm. Therefore, a common practice is to leave nasal oxygen running after preoxygenation (standard or HFNC) during laryngoscopy.

During intubation, if the first attempt fails, it is imperative to re-oxygenate the patient between attempts. This might involve returning to mask ventilation or inserting a supraglottic airway if mask ventilation is difficult (more on that in subsequent sections). The threshold for pausing and reoxygenating should be low: if the first intubation attempt exceeds 20–30 s or if saturation drops significantly, the intubator should stop, resume ventilation with BVM or SGA with high-flow oxygen until saturations recover, then attempt the next step in the algorithm.

### 4.4. Medication Planning

Choice of induction and paralytic agents can influence airway management success. Rapid Sequence Intubation (RSI) is commonly used in the ICU to minimize aspiration risk and facilitate prompt intubation. RSI involves giving a potent induction agent (e.g., etomidate, propofol, or ketamine) immediately followed by a fast-acting neuromuscular blocker (usually succinylcholine or rocuronium) to rapidly achieve conditions for intubation.

Succinylcholine, while offering the advantage of a rapid onset of action (30–60 s) and a short duration (5–10 min), has significant potential adverse effects that limit its use in the intensive care setting. It acts as a depolarizing neuromuscular blocker by persistently stimulating acetylcholine receptors, causing initial fasciculations followed by paralysis. However, this same mechanism leads to rapid potassium efflux from myocytes, which can precipitate life-threatening hyperkalemia in susceptible patients. These include individuals with prolonged immobilization, burns, trauma, sepsis, neuromuscular disorders (e.g., Guillain–Barré syndrome or spinal cord injury), renal failure, or muscular dystrophies. Furthermore, succinylcholine carries the risk of malignant hyperthermia, bradyarrhythmias, and rhabdomyolysis and is contraindicated in patients with known or suspected myopathies or denervation injuries [19].

Rocuronium, in contrast, is a non-depolarizing neuromuscular blocking agent with a slightly slower onset of action (1–2 min at a dose of 1.0–1.2 mg/kg) and a longer duration (30–60 min). While its longer duration might be concerning in the event of failed intubation, this disadvantage is offset by the availability of sugammadex, a selective relaxant binding agent that can reverse neuromuscular blockade within minutes. Rocuronium is hemodynamically neutral, lacks the risk of hyperkalemia or malignant hyperthermia, and is considered safer in critically ill patients who commonly present with the same contraindications that limit succinylcholine’s use. Its pharmacokinetic profile makes it especially suited for anticipated or unanticipated difficult airways, where prolonged paralysis may be necessary and rapid reversal is available. For these reasons, most experts and recent guidelines now favor rocuronium as the paralytic of choice in ICU airway management, especially when sugammadex is readily accessible [20].

In a difficult airway scenario, the use of muscle relaxants is debated: paralysis can improve laryngoscopic conditions dramatically (by eliminating reflexes and muscle tone, allowing easier tube passage and better view), but it also removes the patient’s ability to breathe or maintain their airway if intubation fails. Thus, if both intubation and ventilation are anticipated to be difficult, awake intubation (avoiding RSI) is preferred, as mentioned earlier [1]. However, if the difficulty is only with intubation and not ventilation, many guidelines still recommend using neuromuscular blockade even in a “difficult” intubation because the benefits in terms of improved intubating conditions and avoidance of patient movement can outweigh the downsides, provided one is prepared to ventilate with a mask or SGA if intubation fails [3]. DAS guidelines (2015) advocate for using neuromuscular blockade early in difficult intubation scenarios, on the basis that struggling against a semi-conscious patient often makes matters worse [3].

The induction agent choice should account for patient physiology: e.g., etomidate is often chosen in shock for its hemodynamic stability, ketamine for asthmatics or profound sepsis (for bronchodilation and maintaining blood pressure, though it can raise BP and heart rate), and propofol is titrated carefully or avoided in unstable patients due to its vasodilatory effects. In any case, doses should be adjusted—the concept of “peri-intubation optimization” extends to giving lower induction doses if needed. The priority is to achieve intubating conditions without causing cardiovascular collapse. Having vasopressors ready (e.g., norepinephrine infusion primed or push-dose phenylephrine) is a wise precaution.

For sedation during awake intubation (if that route is taken), one must achieve a balance: enough sedation to tolerate the procedure, but not so much that the patient loses their airway reflexes or becomes apneic. Medications like dexmedetomidine (for cooperative moderate sedation), ketamine (for dissociative state while maintaining breathing), or a remifentanil infusion combined with local anesthetics are techniques used in elective awake fiberoptic intubations. In ICU, ketamine is popular for this purpose because it tends to support blood pressure and respiration while providing dissociative analgesia.

That said, ketamine’s sympathomimetic effects maybe undesirable in patience with ischemic heart disease, aortic dissection, or intracranial pathology where surges and blood pressure or heart rate could be detrimental. Therefore, sedation strategies should be individualized based on patient comorbidities, hemodynamic profile, and procedural goals [21].

Etomidate is frequently favored for hemodynamically unstable patients due to its minimal effect on cardiac output and systemic vascular resistance. Its rapid onset (30–60 s) and short duration make it well-suited for emergent intubation. However, concerns remain over transient adrenal suppression, particularly in patients with sepsis or critical illness, though this has not been definitively shown to worsen outcomes in the context of single-dose use [22].

Propofol provides rapid onset and smooth sedation but is vasodilatory and negatively inotropic, often resulting in hypotension, especially in hypovolemic or elderly patients. It is typically avoided or carefully titrated in unstable patients, though useful in controlled settings where rapid neurologic evaluation is planned after intubation.

Ultimately, individualized agent selection based on the patient’s underlying physiology, anticipated airway difficulty, and available monitoring and support is critical. Pre-intubation planning should include preemptive vasopressor preparation (e.g., push-dose phenylephrine or norepinephrine infusion) and dose adjustments in shock states as part of a broader peri-intubation optimization strategy.

### 4.5. Team and Logistics

An often-underappreciated aspect of preparation is coordinating the team. Assign specific roles: one person (often a respiratory therapist or second physician) to manage the bag-valve mask and oxygenation, another to administer drugs, another to prepare equipment, etc., while the primary intubator focuses on the procedure. Explicitly state plan A (primary approach) and plan B, C, and D to the team before starting.

Additionally, backup personnel should be alerted early. If an anesthesiology consult service or an on-call difficult airway team is available in the hospital, calling them before initiating an elective or semi-elective intubation in ICU is wise if difficulty is anticipated. In a “cannot intubate, cannot ventilate” scenario, having an extra pair of skilled hands or someone ready to do a surgical airway can be lifesaving. Even in the middle of the night, most guidelines advise that when a difficulty is detected or an intubation fails, one should call for help early [3].

In summary, preparation for a difficult airway in the ICU is all about attention to detail and contingency planning. In a crisis, cognitive function narrows—having a checklist or pre-formulated plan is immensely helpful. It is of particular importance to ensure adequate preoxygenation, proper patient positioning, team coordination, and tailored medication dosing before attempting airway management. Anticipating potential difficulties, understanding patient physiology, and being familiar with individualized tools are essential to minimize complications such as cardiovascular collapse or hypoxia. These seemingly minor considerations collectively determine intubation success and hemodynamic stability.

## 5. Airway Management Techniques and Tools

### 5.1. Direct Laryngoscopy

**Figure 4:**
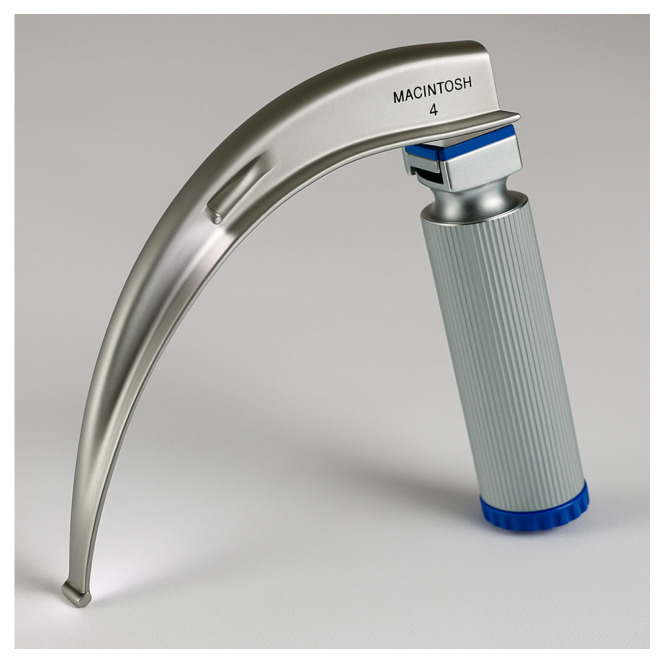
The Macintosh blade: A curved direct laryngoscope blade used for orotracheal intubation.

**Figure 5:**
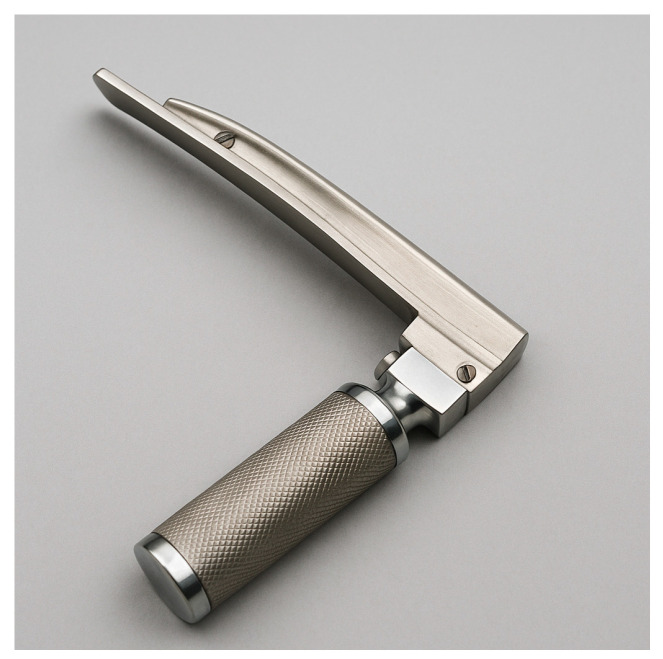
The Miller blade: A straight direct laryngoscope blade, often preferred for pediatric patients and certain adult intubations (e.g., anterior airway).

Direct laryngoscopy (DL) with a handheld laryngoscope (Macintosh (Figure 4) or Miller blade (Figure 5)) has been the foundational technique for endotracheal intubation for decades. The Macintosh curved blade (size 3 or 4 for adults) is most used, inserted into the oropharynx and advanced into the vallecula (space between tongue base and epiglottis) to lift the epiglottis indirectly and expose the vocal cords. The Miller straight blade (size 2 or 3 for adults) is often favored in certain situations (e.g., pediatric patients, or sometimes in adults with a floppy epiglottis) because it is designed to directly lift the epiglottis.

#### 5.1.1. Role in Difficult Airway

DL is typically the first-line approach when a difficult airway is not anticipated (i.e., a “normal” airway). However, even in many difficult airway algorithms, an initial attempt with direct laryngoscopy by a skilled intubator remains part of Plan A, especially if video laryngoscopy is not immediately available or if the operator is particularly experienced in DL. For predicted difficult laryngoscopy, guidelines increasingly recommend using video laryngoscopy (VL) from the outset. There are scenarios where direct laryngoscopy might succeed even if video fails; for example, when the camera lens is obscured by secretions or blood, but a direct line-of-sight could be momentarily obtained with suction and maneuvering. Thus, clinicians should remain adept at DL as a backup to VL and vice versa.

#### 5.1.2. Techniques to Optimize DL

If a direct laryngoscopy attempt yields a poor view (e.g., only epiglottis or only arytenoids visible), several maneuvers can help:

- External Laryngeal Manipulation: Also known as BURP (Backwards, Upwards, Rightward Pressure on the thyroid cartilage) or simply bimanual laryngoscopy (where the intubator uses their right hand to adjust the larynx position externally until a better view is achieved, then an assistant holds that pressure).
- Changing Blade or Size: If a Miller was used, switch to a Mac or vice versa.
- Patient Repositioning: Further optimizing the head elevation or pillow height can improve the angle. During intubation, slight adjustments such as an assistant performing head lift or neck extension can help if not contraindicated.
- Use of an Intubation Adjunct: If a partial view of the glottis is obtained (like seeing arytenoids or epiglottis), a gum elastic bougie can be inserted (feeling for the tracheal rings), even without full view of cords, to serve as a guide for the endotracheal tube
- Limiting Attempts: Evidence has shown that repeated attempts at direct laryngoscopy without changing something are detrimental. Mort (2004) noted that multiple laryngoscopic attempts were associated with a steep rise in complication rates (hypoxia, aspiration, or airway trauma) [23,24]. Most algorithms advise a maximum of 2–3 DL attempts by an experienced operator before moving to another technique [3].

Historically, direct laryngoscopy success depends heavily on operator training and patient factors. In an elective OR setting, success on first attempt is >90% for experienced anesthesiologists, but in the ICU, success rates on first attempt by critical care physicians or trainees can be significantly lower, around 60–80% depending on experience [4]. A large observational study in the ICU found that the incidence of difficult laryngoscopy (Cormack–Lehane grade III or IV view) was about 10–20%, and risk factors mirrored those in OR (obesity, short neck, etc.) [24]. As difficulty increases, adjuncts like bougies become extremely useful. A 2018 randomized trial by Driver et al. in an emergency department setting found that using a bougie on the first attempt in patients with at least one difficult airway characteristic improved first-pass intubation success compared to using a standard stylet [25]. This suggests that integrating such adjuncts early during direct laryngoscopy can be beneficial when difficult intubation is anticipated.

Direct laryngoscopy requires a direct line of sight to the glottis. Thus, any anatomic issue that prevents creating that line (e.g., a large tongue, reduced neck mobility, anterior larynx, or an obstructing mass) can make DL much more technically challenging. It also does not allow others to see what the operator sees, which can make teaching or assisting more challenging. In contrast to video laryngoscopy, DL has a steep learning curve—practice and experience are crucial. In the ICU, practitioners may not intubate as frequently as anesthesiologists, so DL skills can vary widely among operators. Additionally, in an emergency with high-stress, novices may find DL more daunting than using a video screen.

In summary, direct laryngoscopy remains an important technique and is the foundation upon which difficult airway management is built. Mastery of DL is important not only for its own success rate but also because many rescue techniques (like bougie usage) are applied in the context of direct laryngoscopy.

### 5.2. Video Laryngoscopy

**Figure 6:**
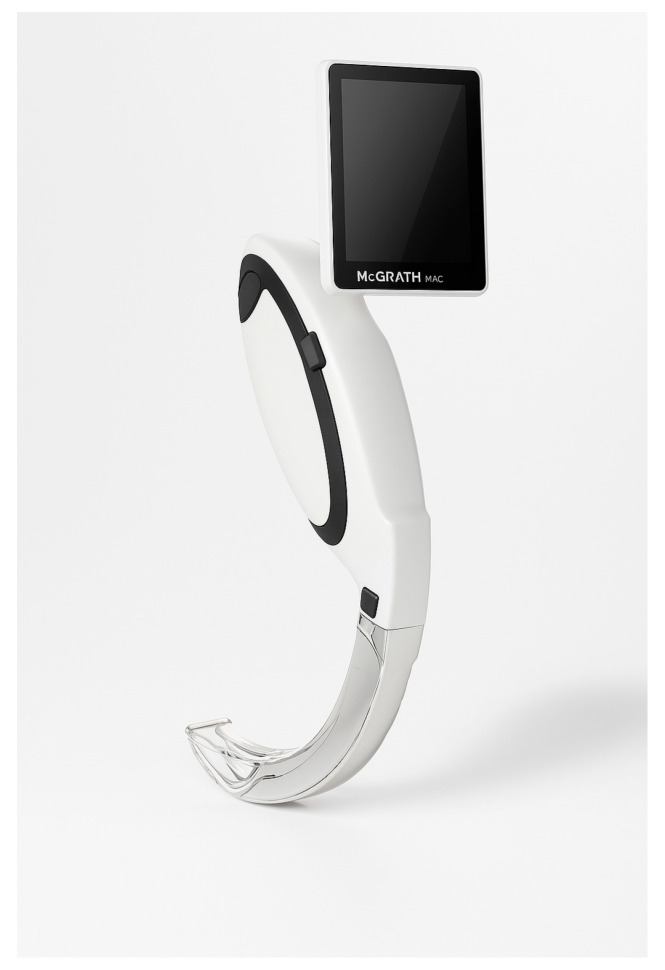
Video laryngoscope blade (McGrath). (Source: Stryker, available at: https://www.stryker.com/us/en/emergency-care/products/mcgrath-mac-ems.html, accessed on 31 May 2025) [27].

Video laryngoscopes (VL) use a camera at or near the tip of the laryngoscope blade to display the glottic view on a screen. There are many devices available (e.g., Glidescope, C-MAC, McGrath (Figure 6), Airtraq, King Vision, etc.), but they generally fall into two categories: those with a Macintosh-like blade that can be used similarly to direct laryngoscopy but with the advantage of video (e.g., C-MAC or McGrath Mac) and those with a hyperangulated blade (like Glidescope, which has a more acute curvature to reach anteriorly). The hyperangulated types often require a preformed rigid stylet to guide the ETT along the blade’s curvature into the trachea. Video laryngoscopy has transformed airway management by often providing a much-improved view of the glottis compared to direct line-of-sight.

Selection of video laryngoscope should not be considered one-size-fits-all. Specific device choice must be tailored to patient anatomy, the predicted degree of difficulty, and operator experience. Macintosh-type VL blades may be preferable in patients with relatively preserved airway alignment (e.g., trauma-free and normal neck mobility), while hyperangulated blades are advantageous in those with cervical spine immobility or morbid obesity where a direct view is anatomically impossible. However, the enhanced view provided by hyperangulated blades can paradoxically make tube advancement more difficult, necessitating specific techniques and tools (e.g., rigid stylet shaping, rotation maneuvers, or use of bougie). Moreover, successful intubation is highly operator-dependent; glottic visualization does not guarantee success unless the provider is well-practiced in manipulating the endotracheal tube with the given blade configuration.

Some VL systems (e.g., Airtraq or King Vision) incorporate built-in intubating channels that facilitate tube guidance. Others, like McGrath and C-MAC, resemble traditional blades more closely and allow for a wider range of manipulation but may require more skill. Thus, blade geometry, intubating channel presence, and familiarity with the device all impact intubation success [26].

#### 5.2.1. Role in Difficult Airway

VL is strongly recommended as a first-line device in many difficult airway scenarios. The DAS 2015 guidelines explicitly mention that video laryngoscopy should be considered early when direct laryngoscopy is difficult or after one failed attempt with DL [3]. The ASA 2022 guidelines also acknowledge the role of VL as an important adjunct that improves visualization and may increase success in difficult intubations [1]. In the ICU, where patients frequently have difficult airway characteristics, some experts advocate using a video laryngoscope for every intubation (even those predicted to be easy) to maximize first-pass success and minimize airway trauma from multiple attempts [4].

The key advantage is improved glottic visualization. The camera can often see “around the corner” of the tongue to an extent that the human eye cannot. This is particularly true in an anterior airway; the hyperangulated blades can make even Cormack–Lehane grade 3 direct views become grade 1 or 2 on video. Additionally, VL allows shared visualization—the person intubating and others in the team can see the screen. Another advantage in the ICU context, as noted in studies, is that video laryngoscopy can improve first-pass success in operators who are not airway experts. A large observational study by Jaber et al. (2019) in ICU patients found that using VL was associated with higher first-pass intubation success and fewer severe complications compared to direct laryngoscopy, especially in scenarios of anticipated difficulty [28].

Multiple trials and meta-analyses have evaluated VL vs. DL. A 2016 Cochrane review by Lewis et al. looked at VL versus DL in thousands of elective surgical patients; VL significantly reduced failed intubations and improved glottic views, particularly in anticipated difficult airways, though it did not always reduce the number of attempts or intubation time in all settings [29]. In the ICU specifically, the MACMAN trial (Lascarrou et al. 2017) was a randomized controlled trial comparing video laryngoscopy (Glidescope) vs. direct laryngoscopy for intubation in critically ill patients [30]. Interestingly, it did not find a statistically significant difference in first-pass success between VL and DL overall (around 67% vs. 70%, *p* = 0.2), possibly due to the learning curve and the fact that not all patients had difficult airways in that trial. However, the VL group had better glottic visualization (more grade 1 views) and in post hoc analysis seemed beneficial for less experienced intubators. Criticisms included that many operators were not experienced with the Glidescope and were more comfortable with DL, which might have biased the results. Given this evidence, current clinical practice has shifted such that many anesthesiologists and critical care doctors reach for a video laryngoscope first in high-risk scenarios. The learning curve for VL is considered easier in some respects (the view is usually good even for novices), though coordinating hand-eye skills is a new challenge.

#### 5.2.2. Technique Considerations

Using a video laryngoscope is not foolproof. One common pitfall is getting a great view of the cords on the screen but struggling to pass the tube. This often happens with hyperangulated blades: the angle from mouth to glottis is so acute that advancing the tube is difficult. Solutions include the following:

- Using the proprietary stylet that matches the blade curvature (e.g., the rigid stylet that comes with Glidescope, shaped to approximate the blade angle). The tube should be preloaded on this, and often a slight rotation of the tube or withdrawal of stylet as the tip is near cords helps.
- Some video laryngoscopes (like C-MAC D-blade) allow using a bougie instead of a stylet.
- Another approach if intubation is tricky is to slightly withdraw the video laryngoscope blade; counterintuitively, pulling back a little can give more room to maneuver the tube and lessen the sharp angle needed.
- Ensure the patient’s head is not extremely flexed; occasionally, a bad angle can be improved by adjusting head position even during the attempt.

Additionally, blood or vomit in the airway can quickly obscure the camera, making VL temporarily useless until suction clears the view. It is crucial to have suction ready and to use it promptly. Some practitioners will do a quick direct look if they suspect the camera is too soiled, or they might switch to DL in a heavily contaminated airway because our eyes can sometimes see through a thin layer of blood better than the camera can (and wiping a blade is easier than cleaning a camera lens mid-intubation). That said, many modern VL blades are disposable plastic covers that can be quickly swapped if one gets too dirty.

The ASA’s 2022 guidelines mention that video laryngoscopy should be readily available and considered as a primary approach in difficult airway management [1]. The DAS 2015 guidelines incorporate VL as an option in Plan A, and if not used initially, then certainly as part of Plan B for a failed direct laryngoscopy [3].

Cost and availability can be limited in some settings—not every ICU or smaller hospital has a state-of-the-art video scope available 24/7. Additionally, reliance on video can erode practitioners’ DL skills over time, which is a concern if the video fails or is not around. However, that trade-off is generally deemed acceptable given the improved safety. Importantly, video laryngoscopy is not a substitute for proper technique—poor technique will still result in failure. For instance, if an operator does not adequately clear the tongue out of the way or insert the blade properly, they might not see anything, even with a camera. Training is needed to use VL effectively.

In summary, video laryngoscopy represents a transformative tool in airway management but must be applied thoughtfully. Device choice should reflect anatomical and contextual factors, and while VL improves glottic visualization compared to direct laryngoscopy, successful intubation still hinges on appropriate technique and operator familiarity. Understanding the distinctions between standard and hyperangulated blades, the implications of intubation channel presence, and the anticipated difficulty of the airway is essential to optimize outcomes with VL.

### 5.3. Supraglottic Airway Devices (SGAs)

**Figure 7:**
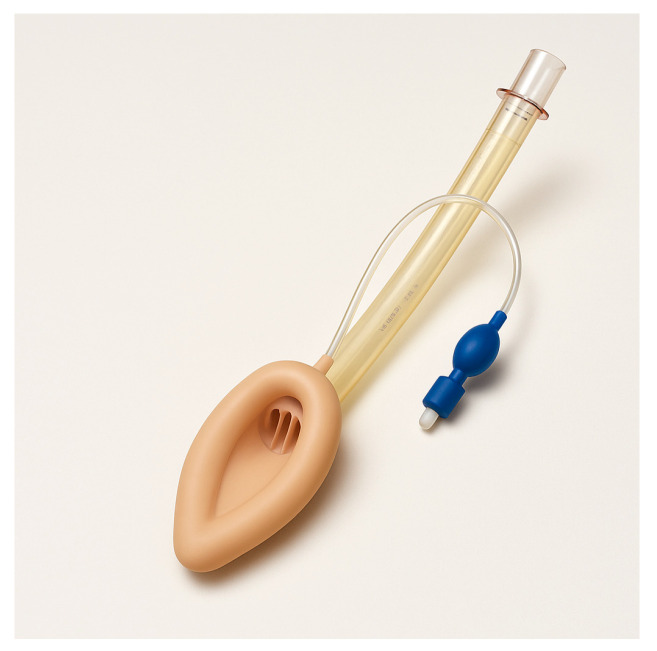
Classic Laryngeal Mask Airway (LMA): An inflatable supraglottic airway device.

**Figure 8:**
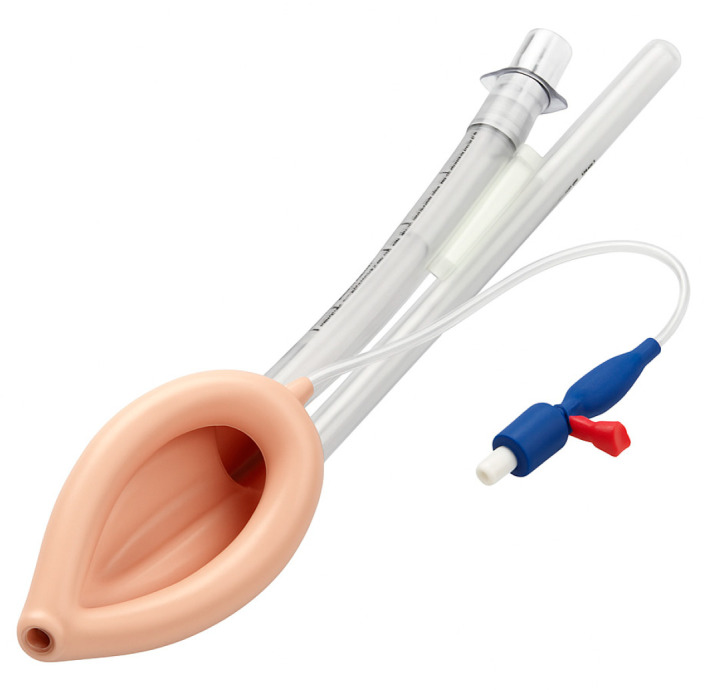
Laryngeal Mask Airway ProSeal: Featuring a dorsal cuff for improved seal and aspiration protection.

**Figure 9:**
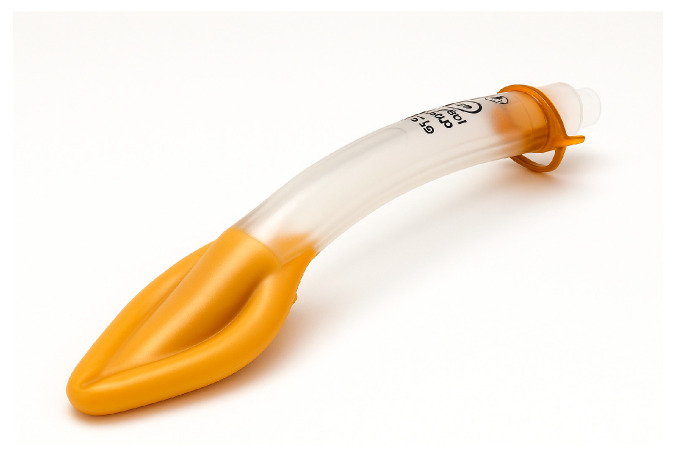
i-gel supraglottic airway.

Supraglottic airway devices sit above the glottis and allow ventilation without passing a tube through the vocal cords. The classic example is the Laryngeal Mask Airway (LMA), but there are many variants (Table 1): LMA Classic (Figure 7), LMA ProSeal (with a gastric port) (Figure 8), LMA Supreme, i-gel (Figure 9) (a gel-like cuffless device), etc. These devices are inserted blindly into the pharynx where they seal around the laryngeal inlet. They provide a hands-free way to ventilate the lungs, delivering oxygen and anesthetic gases if needed. In elective anesthesia, LMAs are commonly used for routine airway management in appropriate cases. In the context of a difficult airway, SGAs serve primarily as a rescue device for ventilation when intubation fails or as a conduit for intubation in some situations.

#### 5.3.1. Role in Difficult Airway

Nearly all difficult airway algorithms incorporate SGAs as part of the plan when face-mask ventilation or intubation is unsuccessful. For example, the DAS 2015 guidelines designate insertion of an SGA as Plan B after failed intubation attempts to “buy time” and oxygenate the patient [3]. The ASA guidelines also suggest that if initial intubation efforts fail, placing a supraglottic airway can establish ventilation and should be considered before moving to surgical access (provided the patient can be ventilated through the SGA) [1]. In an ICU or emergency scenario, an SGA (like an LMA) can be lifesaving if one cannot intubate and cannot ventilate with a bag-mask.

#### 5.3.2. Use as Rescue Ventilation

If at any point the patient cannot be ventilated with a bag-mask (like severe obstruction or operator difficulty) and intubation is not yet achieved, an SGA should be attempted. Devices like the i-gel or LMA Supreme are favored in emergencies because they are relatively easy and quick to insert (the i-gel has no cuff to inflate, simplifying insertion). Success rates of SGAs in establishing ventilation in “cannot intubate, cannot ventilate” (CICV) scenarios are high. For example, a study by Thomsen et al. found that use of a supraglottic airway as a primary rescue device following failed tracheal intubation was successful in 93.1% of cases [31]. NAP4 documented multiple instances where an LMA saved the day in unanticipated CICV scenarios, allowing oxygenation where intubation failed, although it also noted that in some CICV cases, SGA insertion was not attempted when it should have been.

#### 5.3.3. Specialized SGAs for Intubation

**Figure 10:**
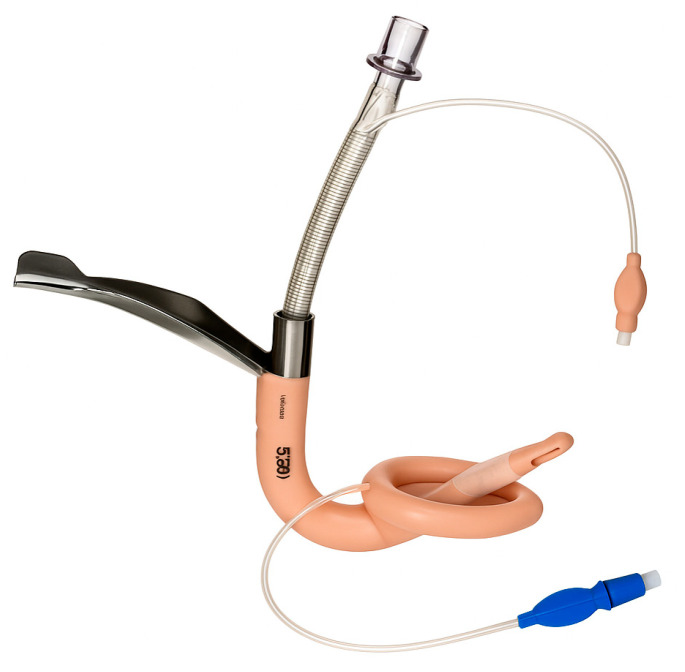
Fastrach Laryngeal Mask Airway.

Some supraglottic devices are designed to facilitate intubation. The LMA Fastrach (Figure 10) (also called Intubating LMA, ILMA) has a rigid airway tube that can guide an endotracheal tube into the trachea blindly. One can ventilate through the ILMA and pass a specially designed ETT through it. More commonly now, rather than blind, a fiberoptic scope can be inserted through an LMA to achieve intubation. For example, if an LMA (like a ProSeal or i-gel) is in place and ventilating, one can thread a fiberoptic bronchoscope through the LMA, visualize the cords and advance into the trachea, then railroad an ETT over the bronchoscope. This is an elegant solution to a difficult airway: ventilate first, then intubate through the ventilating device. However, it requires that an SGA can be placed and that ventilation is adequate through it.

The strength of SGAs is the ease of insertion (much easier than intubation, generally) and the ability to oxygenate/ventilate without needing perfect visualization of anatomy. They also relieve the provider from the task of maintaining a mask seal and airway maneuvers, thus freeing hands and attention for other things once placed (like preparing for intubation or cricothyrotomy if needed). Second-generation SGAs (ProSeal, i-gel, LMA Supreme) are preferable in difficult airways because they have a gastric drainage port to vent passively regurgitated stomach contents, theoretically reducing aspiration risk, and they often provide a higher seal pressure allowing for more effective ventilation at higher pressures.

#### 5.3.4. Limitations of SGAs

The main limitation of SGAs in an emergency is that they do not secure the airway against aspiration. In the ICU, most patients are non-fasting and often at high risk for aspiration. If an SGA is used, aspiration can still occur around the device if the patient vomits, and any ventilation is likely pushing air into the stomach as well (especially if positioning or technique are not ideal). If the choice is between immediate death from hypoxia or a risk of aspiration, oxygenation takes priority. Many guidelines emphasize that risk of aspiration should not deter using an SGA in a cannot intubate/cannot ventilate scenario—oxygenation is paramount; aspiration if it happens can be managed with suction and later care, but anoxic brain injury is irreversible.

Another limitation is that ventilation may be suboptimal if the patient’s lungs are very difficult to ventilate (e.g., severe ARDS requiring high pressures might leak). If peak inspiratory pressures above 30 cm H_2_O are needed, an SGA may leak significantly. Additionally, SGAs might not sit properly in patients with altered airway anatomy (distorted pharynx by tumor or infection). In such cases, insertion might fail or the seal might never form. In elective practice, success rates of LMAs are well over 95% for ventilation. In emergency use, data is mostly observational. For example, in NAP4, among CICV cases, failure to oxygenate with an SGA occurred in some instances, especially if the obstruction was at the laryngeal level or below. However, in many cases where an SGA was attempted, it either restored oxygenation or at least allowed partial ventilation bridging to a cricothyrotomy [7].

The DAS algorithm’s Plan B is basically “attempt ventilation with a supraglottic airway” [3]. If this works, they then consider either waking the patient (if it was an elective case) or intubating through the SGA or proceeding with surgery if feasible on SGA. In ICU, waking up is rarely an option because the patient is likely to need the airway for critical illness. As such, if an SGA is placed and working, one can consider it a temporary lifesaver but usually will proceed to secure the airway definitively. That could mean intubating through the SGA, as described earlier, or if that fails and the patient cannot be woken, proceeding to cricothyrotomy (Plan D). ASA guidelines similarly include LMAs as an intermediate step for ventilation [1].

In summary, supraglottic airway devices (SGAs) serve a vital role as both primary and rescue airway tools, with clinical utility varying based on patient risk factors and procedural context. First-generation SGAs, such as the LMA Classic, provide basic supraglottic ventilation and are generally appropriate for low-risk, elective cases—particularly in fasting patients without elevated intra-abdominal pressures or aspiration risk. However, their lack of a gastric drainage port and limited oropharyngeal seal pressure make them less suitable in critically ill or emergency settings. In contrast, second-generation SGAs such as the LMA ProSeal, LMA Supreme and i-gel incorporate design enhancements including higher sealing pressures (allowing better ventilation at elevated airway resistance), integrated gastric drainage channels (reducing aspiration risk), and anatomically contoured or cuffless profiles for easier and more rapid insertion. These features make them preferable in patients requiring positive pressure ventilation (e.g., obese patients, ARDS, or patients in lateral/prone positions), or those at moderate aspiration risk (e.g., pregnancy, hiatal hernia, gastroparesis, or gastroesophageal reflux disease). Notably, the i-gel is often favored in emergency and ICU settings due to its fast, intuitive insertion, reliable seal, and lack of cuff inflation requirement. While SGAs are not definitive airway tools, they remain essential bridging devices—restoring oxygenation in “cannot intubate, cannot ventilate” (CICV) scenarios and often allowing subsequent fiberoptic-guided intubation or safe transition to a surgical airway when needed [32,33].

### 5.4. Flexible Fiberoptic Intubation

**Figure 11:**
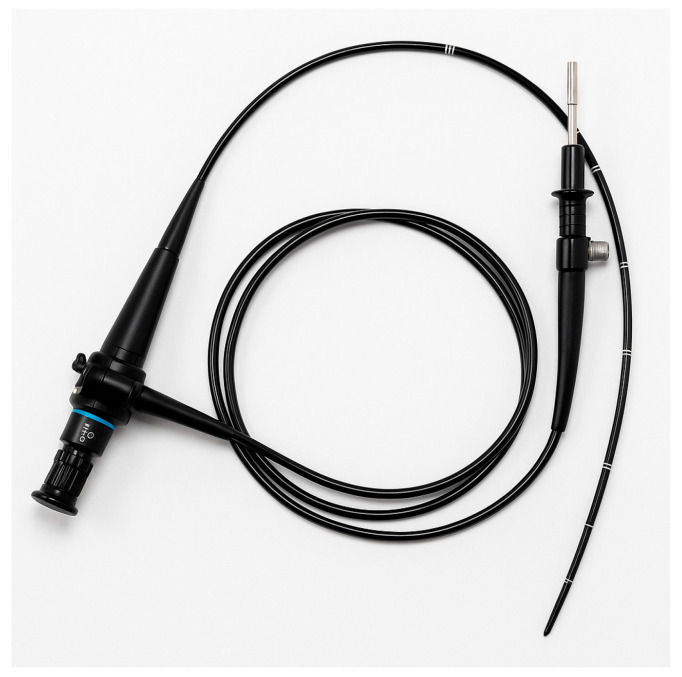
Commercial fiberoptic intubation scope.

Flexible fiberoptic intubation involves using a fiberoptic bronchoscope (a thin, camera-equipped flexible scope) to navigate through the airway to the trachea and then advancing an endotracheal tube over the scope into the trachea (Figure 11). It is often performed with the patient awake or lightly sedated after thorough topical anesthesia of the airway, especially when difficulty is anticipated. Fiberoptic intubation can be done orally or nasally.

#### 5.4.1. Role in Difficult Airway

Fiberoptic bronchoscopy has long been considered the gold standard for managing an anticipated difficult airway, particularly when the patient can maintain spontaneous breathing. If one expects that direct laryngoscopy will be nearly impossible (for example, in severe cervical spine deformity, mouth opening <1 cm, or big tumors in the oropharynx), an awake fiberoptic intubation (AFOI) is often the safest approach. The patient’s own respirations ensure oxygenation while the operator uses the fiberoptic scope to visualize the vocal cords and pass the tube. ASA guidelines have consistently recommended awake intubation via fiberoptic scope as a primary plan for anticipated difficult airways [1].

Fiberoptic intubation offers many advantages. It can be done with the patient awake, breathing spontaneously, thereby avoiding the risk of apnea in a difficult airway. It can navigate through nasal or oral routes, around obstacles. It also allows suctioning via its working channel and insufflation of oxygen through the scope if needed. It is also useful in scenarios of anatomically difficult intubation but where ventilation is fine. It is one of the few techniques that can be used when mouth opening is severely limited. Additionally, fiberoptic scopes can be passed through supraglottic devices as mentioned earlier, aiding a stepwise approach.

The success of fiberoptic intubation heavily depends on operator skill and patient preparation. In skilled hands, success rates are very high. Studies show success rates of ~90–100% for awake fiberoptic intubation in patients with predicted difficult airways when performed by experienced anesthesiologists. A randomized trial by Rosenstock et al. (2012) compared awake fiberoptic intubation with awake video laryngoscope intubation in anticipated difficult airways and found both had similarly high success rates, with fiberoptic being slightly slower in some cases [34]. Complication rates like severe hypoxemia or aspiration during AFOI are very low in elective settings.

In ICU or emergencies, data is sparser, but there are case reports. A case report in a critical care setting noted that awake fiberoptic achieved intubation without incident in a patient with limited mouth opening [35]. The time to intubate is a consideration—fiberoptic intubation can be relatively slow, especially if anatomy is hard to navigate or the patient coughs a lot. Thus, it is not often used in acute emergent “crash” airways. Instead, fiberoptic is ideal for controlled difficult airway management where one has a few minutes to set up and the patient is stable enough to cooperate or at least not crashing.

#### 5.4.2. Techniques

For successful fiberoptic intubation, patient preparation is key. Topical anesthesia of the airway (lidocaine spray to the tongue and oropharynx, or nebulized lidocaine) will blunt gag and cough reflexes. In ICU, patients might already be obtunded, or intubation may be urgent, limiting how much preparation can be done, but even in an obtunded patient, applying lidocaine to the airway can reduce coughing and laryngospasm on instrument insertion.

Sedation is not merely “light” but should be appropriately titrated to ensure patient comfort, reduce procedural stress, and maintain spontaneous ventilation. Insufficient sedation may provoke coughing, retching, or movement that impairs fiberoptic navigation. Thus, sedation should ideally begin before topicalization begins and be balanced to preserve airway reflexes while reducing gag, cough, and sympathetic response. Commonly used agents include dexmedetomidine (which provides cooperative sedation), low-dose remifentanil, or ketamine in selected patients. In addition, premedication with atropine (0.3–0.6 mg IV) may be considered when excessive airway secretions are anticipated, as it can help improve visualization during scope passage [36].

Topical anesthesia helps blunt reflexes (e.g., spraying lidocaine on oropharynx, nebulized lidocaine, or transtracheal injection), but it is the combination of proper sedation and local anesthesia that reduces coughing, nausea, and bronchospasm. Importantly, the tracheal carina is densely innervated with cough receptors; its mechanical stimulation by the fiberscope tip or secretions can provoke a strong cough reflex. Therefore, once the carina is visualized to confirm correct scope placement, contact should be minimized or avoided altogether [37].

During AFOI, oxygen can be given via nasal cannula or special atomization devices, since the patient is breathing spontaneously. The scope is gently advanced: first through the oropharynx, identifying landmarks (uvula and epiglottis), and then the glottis is identified and the scope passed between the vocal cords into the trachea. Once carina or tracheal rings are seen, confirming placement in the trachea, the endotracheal tube (which is preloaded on the scope) is advanced over the scope into the trachea and the scope is removed.

#### 5.4.3. Limitations of Fiberoptic Intubation

Fiberoptic intubation requires a clear airway to see through the scope. Blood, vomit, or even very heavy secretions can make it extremely challenging, as the tip of the scope can become obscured. Repeatedly cleaning the lens with the little port (spraying saline or using a scope that has a built-in suction/flush) may be necessary. In a bleeding airway (like facial trauma), fiberoptic intubation is often not the best choice due to this. Similarly, if the patient cannot cooperate at all and is moving or bucking, a fiberoptic scope can easily be damaged or just not able to be maneuvered effectively. That is why in combative or severely agitated patients, AFOI is rarely attempted; instead, one might have to do an RSI with double setup for surgical airway. Another limitation is training—many current trainees do fewer fiberoptic intubations because video laryngoscopy has taken center stage for many difficult airways.

ASA emphasizes fiberoptics for anticipated difficult airways, stating an awake intubation via flexible scope is often indicated [1]. DAS guidelines incorporate fiberoptics primarily in the awake intubation context rather than as part of the emergency algorithm after induction. In an already induced difficult airway scenario, a fiberoptic becomes less useful except via an LMA conduit, because if the patient is not breathing and time is short, a fiberoptic is slow.

### 5.5. Other Adjuncts and Techniques

**Figure 12:**
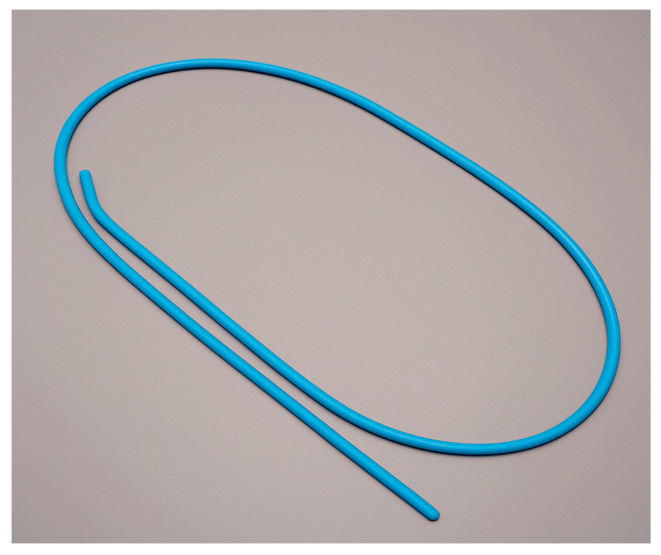
Endotracheal tube introducer/bougie.

In addition to the primary tools above, several adjuncts and special techniques can facilitate airway management in difficult situations:

- Intubation Stylets and Bougies: An intubation stylet is a malleable metal or plastic rod inserted into the ETT to allow it to be shaped and to provide some rigidity. Shaping the ETT to an optimal curve (such as the “hockey stick” with a 35° angle 5 cm from the tip for direct laryngoscopy) helps aim the tube. A bougie (also known as a gum elastic bougie or Frova introducer) is a long, semi-rigid rod often with a coude tip (angled tip) (Figure 12). It is used as a tactile introducer: if one can pass the bougie through the cords or under the epiglottis into the trachea, one can then railroad the ETT over the bougie into the trachea. The DAS 2015 guidelines recommend that if a full view of the glottis is not obtained on the first attempt, a bougie should be used on the next attempt [3]. Many difficult airway algorithms consider the bougie an essential first-line adjunct rather than a rescue.
- While these devices greatly improve intubation success when used appropriately, they also carry a risk of complications if handled improperly. Reported risks include airway trauma such as tracheal or bronchial perforation, vocal cord injury, cricothyroid membrane disruption, laryngeal edema, and bleeding—especially if the device is advanced forcefully or blindly. Bougie-related trauma has been associated with over-insertion (>30 cm), use of excessive force, or railroading the tube over resistance. Similarly, stylets should not protrude beyond the distal end of the tube, must retain a gentle curvature, and should never be forced past resistance. All introducers should be well-lubricated prior to use. For these reasons, bougies and stylets must be used with caution, by trained providers, and with attention to tactile feedback to avoid worsening an already difficult airway situation [38,39].

- Alternate Laryngoscope Blades: Sometimes a change in blade can make intubation possible. For example, if a Mac blade is not giving a view, a Miller blade can directly lift a floppy epiglottis. Devices like the WuScope or Bullard laryngoscope (older rigid fiberoptic laryngoscopes) exist for specific difficult scenarios like very limited mouth opening; they allow indirect visualization like VL but in a thinner profile. These are less common nowadays with widespread VL.
- Optical Stylets/Lightwands: A lightwand (e.g., Trachlight) is a semi-rigid stylet that produces a bright light at the tip. In a darkened room, when the stylet with an ETT is advanced into the trachea, one can see a well-defined glow in the neck (if in trachea) versus a more diffuse glow if in the esophagus. This technique of transillumination can facilitate blind intubation, particularly useful in situations of blood where you cannot see well, though its use has waned. Optical stylets (like the Bonfils) have an eyepiece or camera on a rigid stylet that can be used to see the glottis through the mouth with minimal opening.
- Cricothyroid Membrane Puncture and Jet Ventilation: As an emergency temporizing measure, one can puncture the cricothyroid membrane with a large-bore needle (14G or so) and connect to a jet insufflation device or even a makeshift adapter to provide oxygenation (needle cricothyrotomy). This is part of some emergency algorithms, especially for children under 8 where a surgical cric is often not recommended due to small anatomies. However, needle jet ventilation provides oxygenation but not ventilation; CO_2_ removal is inadequate, buying perhaps 30–45 min at most before acidosis and hypercapnia become critical (in an adult). It is mainly to keep O_2_ saturation up while preparing for a definitive airway. In adults, most algorithms now favor going straight to surgical cric rather than needle, as needle cric had high failure and complication rates in past audits [7].
- Retrograde Intubation: A rarely used technique where a needle is passed through the cricothyroid membrane, a wire is threaded up out the mouth or nose, then an ETT is threaded over the wire from above and guided into the trachea as the wire is pulled down. It is a slow process and almost never used in modern practice with other tools available.
- Positioning Adjuncts: As mentioned, ramping obese patients is critical. Additionally, head-elevated laryngoscopy position (HELP) is encouraged in obesity, which is basically the ramp position. Using a sternal plunge or towel under the shoulders in infants (due to large occiput) is another example of simple adjustments to aid intubation.
- Cricoid Pressure (Sellick’s maneuver): Often used during RSI to reduce passive regurgitation risk by compressing the esophagus. However, cricoid pressure can worsen laryngoscopic view or make intubation harder by distorting the anatomy. The IRIS trial (2019) investigated cricoid vs sham in RSI and found no significant difference in aspiration rates, but intubation was slightly more difficult in the cricoid group [40]. Many guidelines now make cricoid pressure optional and say to release it if it impedes intubation [2]. As such, in a difficult airway scenario, one should be willing to ease or let go of cricoid pressure if it is affecting the view or tube passage.
- High-flow nasal oxygen during apnea: Although described in preoxygenation, it is worth noting again as an adjunct during intubation—nasal oxygen can be left in place to prolong the safe apnea duration.
- Vocal cord manipulation: Sometimes, even with a good view, the tube will not go in because it keeps hitting arytenoids or aryepiglottic folds. A maneuver known as bimanual laryngoscopy (intubator uses right hand to adjust the larynx) or having an assistant use a gloved finger or external pressure to move a floppy epiglottis can guide the tube in.
- Confirmation Adjuncts: After intubation, aside from capnography, devices like an esophageal detector device (an aspirating bulb that does not reinflate if in esophagus) exist, but end-tidal CO_2_ is standard.

### 5.6. The Surgical Airway (Cricothyrotomy)

When all else fails or when anatomic factors preclude non-surgical options, obtaining a surgical airway is the final step in difficult airway management. Cricothyrotomy (cricothyroidotomy) is the procedure of establishing an airway by making an incision through the cricothyroid membrane and inserting a tube (endotracheal or tracheostomy tube) directly into the trachea. This bypasses upper airway obstructions or swelling. It is the definitive “Plan D” in the standard difficult airway algorithm—often summarized as the CICV or CICO situation: “cannot intubate, cannot oxygenate,” meaning attempts at intubation and ventilation have failed and the patient’s oxygenation is in peril.

#### 5.6.1. Indications

Cricothyrotomy is indicated in an emergency when you cannot secure the airway from above and the patient cannot be adequately oxygenated by any other means (mask, SGA, etc.). Examples include severe facial trauma where intubation is impossible, upper airway obstruction (e.g., anaphylactic swelling or a big tumor) that blocks even SGA insertion, or any scenario where intubation attempts have failed, and bag-mask ventilation is ineffective. Some specific situations might prompt going to cricothyrotomy early, such as massive maxillofacial trauma with hemorrhage where visualizing landmarks is impractical—a surgical airway might be the primary approach rather than trying intubation.

#### 5.6.2. Procedure:

There are a few techniques:

- Surgical (scalpel–bougie) technique: This is the technique advocated by many modern algorithms. It involves a vertical skin incision over the cricothyroid membrane region, then a horizontal stab incision through the membrane. The operator then inserts the handle of the scalpel or a finger to keep the opening, slides a bougie into the trachea through the incision, then railroads a cuffed endotracheal tube (usually size 6.0) over the bougie into the trachea, inflates the cuff, and ventilates [3].
- Needle cricothyrotomy with jet ventilation: A large-bore (10–14G) IV catheter is inserted through the cricothyroid membrane at a 45-degree angle caudally. Once air is aspirated, the catheter is advanced and the needle removed. Then, a high-pressure oxygen source is used to insufflate oxygen. This is an emergency oxygenation method, not providing adequate CO_2_ removal. It is mainly for children under 8 (where surgical cric is challenging due to anatomy) or as a very brief bridge in adults if one absolutely cannot do a surgical cric. However, complications include barotrauma, and it requires a specialized setup to do effectively. Therefore, it is generally considered only if there is no other option.
- Percutaneous dilational cricothyrotomy kits: There are kits that mirror the Seldinger technique used in tracheostomies. For example, the Melker kit involves needle puncture, guidewire insertion, and then dilation and inserting a pre-mounted small, cuffed tube. Some providers might use these kits, especially if they are more comfortable with Seldinger techniques.
- Ultrasound can play a valuable role in both planning and performing cricothyrotomy, especially in patients with difficult anatomy. In elective or semi-urgent settings, preprocedural ultrasound can be used to identify the cricothyroid membrane (CTM), which may be challenging to palpate in individuals with obesity, neck trauma, prior surgery, or distorted anatomy. Studies have shown that ultrasound localization of the CTM is faster and more accurate than palpation, particularly in female and obese patients [41].

#### 5.6.3. Success and Training

Cricothyrotomy success rates in an emergency vary, but in skilled hands they are generally high (~90% in field trauma scenarios by trained personnel) [42]. NAP4 found that emergency surgical airways were attempted in several cases; some were too late or were not done at all when indicated, contributing to poor outcomes [7]. When done, many were successful, but a few attempts failed due to technique issues. One big issue is a lack of training—many physicians have never performed a cricothyrotomy on a patient, so when the moment comes, hesitation or error can occur. This is why regular simulation training and familiarity with one’s cricothyrotomy kit is important.

#### 5.6.4. Complications

Even when successful, cricothyrotomy can have complications, such as bleeding. Fortunately, the cricothyroid area is relatively avascular compared to a formal tracheostomy site. Misplacement or later subglottic stenosis are also potential complications. However, these are acceptable risks in a ‘cannot oxygenate’ scenario. Improper technique can lead to injury of the larynx or posterior tracheal wall. If the incision is too high (through thyroid cartilage), it can damage vocal cords; too low and one might go through cricoid or first tracheal ring (essentially a tracheostomy). The cricothyroid membrane is the ideal spot because it is superficial and safe.

Once a cricothyrotomy is done and the patient stabilizes, typically it needs to be converted to a formal tracheostomy or definitive airway by an experienced surgeon within 24 h. Guidelines from the DAS (Plan D) and ASA emphasize that a surgical airway should not be delayed once it is clear it is a CICO situation [1,3]. They advise a maximum of perhaps four failed attempts (including SGA attempts) before moving on, or if at any point oxygen saturation is critically low and falling, to transition to a surgical airway even if not all algorithm steps are exhausted.

## 6. Confirmation of Airway Placement

Ensuring correct placement of airway devices, especially endotracheal tubes (ETTs), is critical to avoid devastating complications such as esophageal intubation, hypoxia, and cardiac arrest. Both the American Society of Anesthesiologists (ASA) and Difficult Airway Society (DAS) guidelines emphasize that confirmation of tracheal placement must be immediate, reliable, and repeatable [1].

The gold standard for confirming ETT placement is continuous waveform capnography, which displays a square-shaped end-tidal carbon dioxide (ETCO_2_) trace throughout the respiratory cycle. A persistent ETCO_2_ waveform with a minimum of three consistent breaths is considered definitive evidence of tracheal intubation in patients with spontaneous or assisted circulation. This method is vastly superior to isolated colorimetric CO_2_ detectors, which can yield false negatives in cardiac arrest or low-flow states [43].

In scenarios such as cardiac arrest, where pulmonary perfusion is reduced, capnography may be falsely negative. In such cases, adjunctive techniques become essential, including the following:

- Direct visualization of the tube passing through the vocal cords during laryngoscopy;
- Symmetrical chest rise and fall;
- Misting or condensation in the tube (less reliable but still used);
- Chest X-ray post intubation;
- Point-of-care airway ultrasound, which has emerged as a rapid, non-invasive tool for confirming tracheal versus esophageal placement.

Ultrasound confirmation typically involves placing a linear probe in a transverse position above the suprasternal notch. Tracheal intubation yields a single air–mucosal interface (“bullet sign”), whereas esophageal intubation produces the “double tract sign” indicating esophageal shadow lateral to the trachea. Ultrasound may also be used dynamically to detect diaphragm movement or lung sliding as evidence of effective ventilation [44].

For supraglottic airway devices (SGAs) like the LMA or i-gel, confirmation is typically clinical—effective chest rise, capnography, ventilator waveforms, and auscultation. However, fiberoptic bronchoscopy through the device can assess for glottic alignment, especially if ventilation is suboptimal or if intubation through the SGA is planned.

The National Audit Project 4 (NAP4) revealed that delayed or failed recognition of misplaced airway devices was a major contributor to morbidity and mortality in airway-related events, reinforcing the importance of objective confirmation. ASA guidelines also note that no device should be assumed to be in the correct position based on clinical impression alone [1,7].

Ultimately, confirming airway device placement is not a single maneuver but a multimodal process that should always include waveform capnography (when available) and clinical correlation. In emergency or ICU settings, rechecking the tube after any patient movement, desaturation, or change in ventilation is critical to prevent “unrecognized esophageal intubation”, which remains a leading cause of airway-related mortality.

## 7. Stepwise Approaches to Difficult Airway Management

Difficult airway management is best executed via an algorithmic approach, which is a predetermined sequence of actions that the team follows in the event of difficulty. Algorithms serve as cognitive aids, reducing the decision-making burden in a crisis and ensuring no critical steps (like calling for help or ensuring oxygenation) are omitted. Several algorithms exist, including those by ASA, DAS, and others, but they share common elements.

### 7.1. General Difficult Airway Algorithm (Plan A, B, C, and D)

#### 7.1.1. Plan A

Initial intubation attempt(s)—The first step is typically a planned attempt at endotracheal intubation under optimal conditions. If difficulty was predicted, this may be done awake (maintaining spontaneous breathing) using fiberoptic or video laryngoscopy. If difficulty was not predicted, this could be an RSI with direct or video laryngoscopy. Plan A includes a limited number of attempts, usually no more than three, and ideally by the most experienced operator available. During Plan A, the following actions should be taken:

- Optimize each attempt (positioning, external laryngeal manipulation, use of bougie on second attempt if first fails, and switch device if needed such as from DL to VL);
- If the first attempt fails, call for help (if not already present)—e.g., an anesthesia stat call or another experienced practitioner;
- Ensure continued oxygenation between attempts;
- Be mindful of time; if intubation is not successful after 2–3 attempts or if saturations are dropping, declare failure of Plan A.

#### 7.1.2. Plan B

Ventilation with supraglottic airway—If intubation fails, the next step is to maintain oxygenation. Typically, this is where one inserts a supraglottic airway (LMA or similar) if mask ventilation is not adequate or not reliable. If mask ventilation is easy and oxygenation is fine, one could theoretically wake the patient up (if elective). In an ICU scenario, you generally cannot just wake the patient, so the focus is on oxygenation. Plan B actions are as follows:

- Insert an appropriate size SGA promptly. Confirm ventilation with chest rise and capnography.
- If the SGA is effective, you have now temporized the situation. At this point, you have options: In the OR elective case, you might wake the patient up or proceed with surgery on LMA if that is acceptable. In ICU, one likely still needs a definitive airway, so another attempt at intubation through the SGA (using fiberoptic scope through the LMA) can be made, or the SGA can be used as a bridge to a surgical airway if intubation cannot be achieved through it.
- If SGA insertion fails or does not provide adequate ventilation), move rapidly to Plan C.

#### 7.1.3. Plan C

Emergency ventilation/“last attempts”—In some algorithms, Plan C is described as trying to oxygenate by any possible means, including resuming mask ventilation. Essentially, ensure oxygenation by mask if LMA has failed, perhaps by trying a two-person BVM technique, or a different SGA. Plan C in OR algorithms often contemplates awakening the patient if possible. In ICU, Plan C is often abbreviated because one usually cannot wake the patient, and if both intubation and SGA have failed, the mask likely was already difficult. Therefore, practically, Plan C in the ICU is recognizing the now-present CICO scenario and preparing for Plan D. However, one last attempt could be performed:

- Try one more attempt at bag-mask ventilation with adjuncts (oral airway, two-person technique);
- Consider a different SGA or reattempting SGA placement;
- This phase should be very brief; if oxygenation is failing, do not persist here long—move to Plan D.

#### 7.1.4. Plan D

Emergency surgical airway—This is the final step: perform a cricothyrotomy to secure the airway surgically. All guidelines converge on this step when noninvasive methods fail. This is where, in an ideal scenario, the difficult airway cart’s surgical set is already open and someone is ready to cut. Use the chosen technique (scalpel–bougie is recommended). Once the cric tube is in place, ventilate and confirm with etCO_2_, and secure the tube. One should prepare for possible surgical airway earlier in the algorithm (during Plan B or C), such that by the time Plan D is reached, minimal additional time is needed to start the procedure. This general flow ensures an organized progression from noninvasive to invasive methods, always emphasizing oxygenation.

## 8. The Vortex Approach

**Figure 13:**
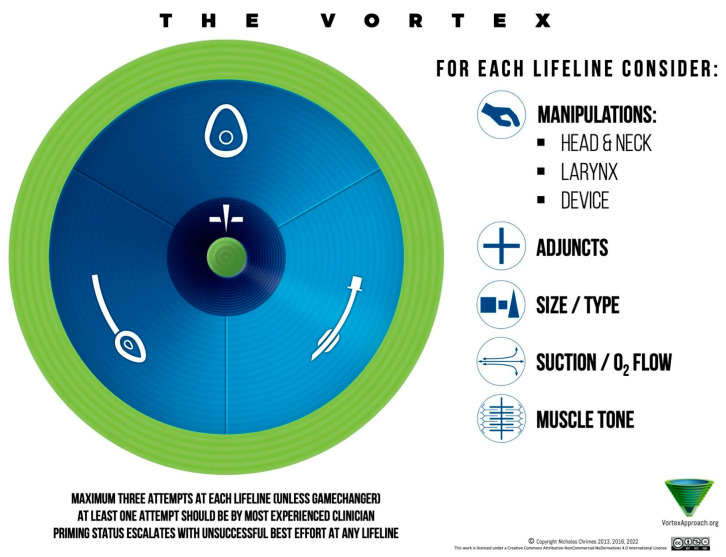
The Vortex approach to difficult airway management (source: The Vortex Approach to Airway Management, available at: https://www.vortexapproach.org, accessed 31 May 2025) [46].

Though not a formal society guideline, the Vortex cognitive aid is worth mentioning. It is an Australian-conceived approach depicted as a spiral: the three non-surgical techniques (face mask, SGA, and intubation) are the sides of a triangle, and the idea is that you rapidly cycles through these attempts (optimizing each) to establish ventilation. If none work after these attempts, you have reached the Vortex’s “green zone” which narrows to the bottom, indicating CICO‚ and you perform cricothyrotomy [45]. The Vortex aid emphasizes visualization (a colored vortex diagram) and human factors (e.g., in a crisis, having a visual cue that time is nearly up can prompt earlier cric) (Figure 13).

## 9. Extubation Planning

ASA and other professional societies stress that when we have a patient who is difficult to intubate, when it is time to extubate, we should do so carefully with a plan to re-intubate if needed. This may involve doing the extubation while the patient is still very awake, maintaining reflexes, and using devices like an airway exchange catheter (a hollow tube left in place through the vocal cords as we remove the ETT, so that if the patient fails extubation or needs reintubation, we can ventilate through it or use it as a guide to rail a new tube back in).

## 10. Limitations

Limitations of this review include its narrative format, which may lack the methodological rigor of a systematic review. Although this article synthesizes current guidelines and key studies, it does not provide a quantitative meta-analysis of outcomes. Additionally, while this review focuses on ICU airway management, the heterogeneity of practice settings and operator skill levels limits generalizability. Finally, device performance data (e.g., success rates of video laryngoscopy or supraglottic airways) may be influenced by local availability, operator experience, and patient anatomy, which may not be uniformly captured in the cited literature.

Future research should focus on high-quality trials comparing first-pass success and complication rates across different devices in critically ill patients, as well as standardized training protocols for non-anesthesiologists managing ICU airways.

## 11. Conclusions

Airway management in critical care is a complex mix of preparation, skill, teamwork, and decision-making under pressure. A difficult airway can turn into a catastrophe within minutes if not managed properly, which is why standardized algorithms and strategies are essential. Each tool has its place in the armamentarium, and the best outcome is achieved when the clinician chooses the right tool at the right time.

Modern guidelines—ASA 2022, DAS 2015, and others—provide a safety net of recommendations distilled from analysis of mishaps (like NAP4) and evidence from studies (like the INTUBE observational study). They emphasize early identification of a difficult airway, thorough preparation (including preoxygenation and positioning), and a stepwise approach that prioritizes oxygenation at every juncture. The concept of “going from Plan A to Plan D” in a timely fashion cannot be overemphasized—delay or repeated unsuccessful attempts are dangerous.

While complications are often unavoidable in anatomically or physiologically difficult airways, they should not occur due to poor anticipation, inexperience, or failure to apply basic principles. The best outcomes are achieved not only through proper device selection, but when airway management is performed by clinicians experienced in handling difficult airways. Each attempt matters and repeated failures worsen outcomes and should be avoided through early escalation and adherence to structured protocols.

In critical care, every second counts and there may be no immediate backup like in the OR. By adhering to the principles outlined in this review, critical care specialists can greatly improve patient outcomes. The literature and guidelines collectively show that when a difficult airway is managed in a structured way, complications are reduced.

## Figures and Tables

**Table 1:** Variants of the Laryngeal Mask Airway (LMA) and supraglottic airway devices.

Name	Type	Material	Advantages	Disadvantages
LAMA Classic	First generation	Silicone	The original design offers less trauma to the throat and lower risk of breathing issues than an endotracheal tube.	Lower oropharyngeal seal pressure; more expensive to sterilize and maintain.
LAMA ProSeal	Second generation	Silicone	Includes a gastric drain port, integrated bite block, and provides a higher oropharyngeal seal.	Bulky, and folding the mask can block the gastric port.
LAMA Supreme	Second generation	Polyvinyl chloride	Single-use version of the ProSeal with a gastric port for drainage.	Bulky, and folding the mask can also block the gastric port.

## Data Availability

Not applicable.

## Abbreviations

AFOI	awake fiberoptic intubation
ASA	American Society of Anesthesiologists
BURP	Backwards, Upwards, Rightward Pressure (on the thyroid cartilage)
CICO	cannot intubate, cannot oxygenate
CICV	cannot intubate, cannot ventilate
DAS	Difficult Airway Society
DL	direct laryngoscopy
ETT	endotracheal tube
FRC	functional residual capacity
HELP	head-elevated laryngoscopy position
HFNC	high-flow nasal cannula
ICU	intensive care unit
ILMA	Intubating LMA (Laryngeal Mask Airway)
LEMON	mnemonic for airway assessment: Look externally, Evaluate 3-3-2 rule, Mallampati class, Obstruction, Neck mobility
LMA	Laryngeal Mask Airway
MACOCHA	a score to predict difficult intubation in ICU patients
MOANS	mnemonic for predicting difficult bag-mask ventilation: Mask seal, Obesity/Obstruction, Aged, No teeth, Snoring
NAP4	Fourth National Audit Project
NIV	noninvasive ventilation/noninvasive positive-pressure ventilation
PEEP	positive end-expiratory pressure
RODS	mnemonic for predicting difficult supraglottic airway use: Restricted mouth opening, Obstruction, Distorted airway, Stiff lungs
RSI	Rapid Sequence Intubation
SGA	supraglottic airway device
THRIVE	Transnasal Humidified Rapid-Insufflation Ventilatory Exchange
VL	video laryngoscopy/Video laryngoscopes
